# Changes in cerebrospinal fluid and blood plasma levels of IGF-II and its binding proteins in Alzheimer’s disease: an observational study

**DOI:** 10.1186/1471-2377-14-64

**Published:** 2014-04-01

**Authors:** Joakim Hertze, Katarina Nägga, Lennart Minthon, Oskar Hansson

**Affiliations:** 1 Clinical Memory Research Unit, Department of Clinical Sciences, Lund University, Malmö, Sweden; 2 Memory Clinic, Skåne University Hospital, Lund and Malmö, Sweden

**Keywords:** Alzheimer Disease, Dementia, Cerebrospinal fluid, Blood plasma, IGF-I, IGF-II, IGFBP-2, IGFBP-3, Insulin

## Abstract

**Background:**

The Insulin-like Growth Factor (IGF)-related system is implicated in neuroregeneration and cell repair, as well as regulating lifespan. IGF-II, one component of this system, has also been found to affect memory functions in a rat model. In this study we explored changes in the IGF-related system in patients with Alzheimer’s disease (AD), including changes in IGF-II levels.

**Methods:**

We measured blood plasma and cerebrospinal fluid (CSF) levels of IGF-I, IGF-II, IGFBP-2 and IGFBP-3 in 72 healthy controls and 92 patients with AD.

**Results:**

We found significantly lower blood plasma levels of IGF-II and IGFBP-3 in patients with AD, compared with controls. The levels of IGF-II and IGFBP-2 were significantly elevated in the CSF from patients with AD. We also found correlations between established CSF biomarkers for AD (tau and P-tau) and components of the IGF system.

**Conclusions:**

CSF and blood plasma levels of IGF-II and some of its binding proteins are changed in patients with AD. Further investigation into this area may unravel important clues to the nature of this disease.

## Background

Alzheimer’s disease (AD), the most common cause of dementia [1,2], is a heterogeneous condition. Neuropathological hallmarks of the disease include senile plaques containing β-amyloid (Aβ42), and neurofibrillary tangles (NTFs), containing hyper-phosphorylated tau (P-tau). The so-called amyloid hypothesis proposes that skewed amyloid homeostasis promotes the accumulation of Aβ42 in the brain. This is commonly thought to represent the earliest event in the development of AD [3].

The insulin-like growth factor (IGF)-related system is comprised of two ligands, IGF-I and IGF-II, their binding proteins and their cell-surface receptors [4]. Some authors also include insulin and the insulin receptor (IR) with this system [5]. IGF-I is mainly synthesized in the liver as a response to growth hormone (GH), but also in other tissues–including neurons–in an autocrine/paracrine manner, seemingly independent of GH [2]. IGF-II is less well understood than IGF-I. It is expressed in the brain during fetal development, but it is also the most abundantly expressed IGF in the adult central nervous system (CNS) [6], with its highest relative concentration in the hippocampus [7]. Both IGF-I and IGF-II are potent growth-promoting and neuroprotective factors in the human nervous system [7,8].

Three types of cell surface receptors bind the ligands of the IGF-related system. IGF-IR binds IGF-I with high affinity, but also IGF-II and insulin with much lower affinity [9]. IGF-IIR binds IGF-II with high affinity, but also IGF-I with much lower affinity [6,9]. Both IGF-I and IGF-II also bind to the IR [9]. IGF-IR and IR are both tyrosine kinase receptors, while IGF-IIR is identical to the cation-independent mannose-6-phosphate receptor. The structurally distinct IGF-IIR not only transmits a signal when IGF-II binds, but also targets the ligand for endocytosis-mediated lysosomal degradation [10].

In biological fluids, IGF-I and IGF-II are normally bound to carrier proteins, IGFBPs, which extend their half-life and modulate their availability and bioactivity. So far, a family of six binding proteins, IGFBP-1 through 6, has been characterized. Of these six proteins, IGFBP-3 is the most abundant one in the bloodstream, while IGFBP-2 is the most abundant one in the intrathecal space [11,12]. IGFBP-2 is detectable throughout the brain, but particularly so in regions undergoing continuous remodelling, such as the olfactory bulb, the cerebellum and the hippocampus [6,11]. IGFBP-2 binds IGF-II with a moderate preferential affinity over IGF-I [13]. The expression of IGFBP-2 correlates with and complements that of IGF-II [6].

Humans, as well as most mammals, experience a cognitive decline in old age. This was long thought to be caused by neuronal cell loss, but has been found to be more related to impaired neuronal plasticity [14]. As we grow older, levels of GH, IGF-I and IGF-II fall progressively [15,16] and an association between circulating levels of IGF-I and cognitive decline has been described [17-19]. Moreover, reduced mRNA expression of IGF-IR, IGF-IIR and IR, in the brain of patients with AD has been reported, with expression levels decreasing as the disease progresses [20,21]. In a recent study by Chen et al., injecting recombinant IGF-II into the hippocampus greatly enhanced memory retention and reduced forgetting in a rat model. Interestingly, this effect seems to be mediated by the IGF-IIR receptor [7], not the more extensively explored IGF-IR. Furthermore, galantamine, an acetylcholinesterase-inhibiting drug used to ameliorate the symptoms of AD, was shown to increase hippocampal levels of IGF-II in mice [22].

There is also evidence linking the IGF-related system to the clearance of β-amyloid. Reduced IGF signaling protects against behavioral deficits, neuroinflammation and neuronal loss in a transgenic mouse model of AD and this effect was found to be associated with the sequestration of soluble, toxic oligomers [23]. Similar results were found in a different study [24]. On the other hand, elevated serum levels of IGF-I seems to increase the clearance of Aβ42 in the brain of mice [25] and IGF-I has also been shown to protect brain cells from Aβ42-induced neuronal cell death [26-28].

In light of evidence linking the IGF-related system to cognition, as well as several changes observed in AD, we hypothesized that alterations in this system might contribute to the pathological mechanisms underlying this form of dementia. While the role of IGF-I has been explored to some degree in patients with AD, less is known about IGF-II in this context. Indeed, to the best of our knowledge, only one previous study has investigated changes in CSF and blood plasma levels of IGF-II in humans with AD-related pathology. In the present study, we investigated the levels of both IGF-I and IGF-II, as well as levels of IGFBP-2 and IGFBP-3 in CSF and blood plasma in cognitively healthy controls and in patients with a clinical diagnosis of AD.

## Methods

### Study participants

This study was performed at the Memory Clinic of Skåne University Hospital in Malmö, Sweden. CSF and blood plasma samples were obtained from 72 cognitively healthy volunteers and from 92 patients diagnosed with AD. The healthy participants were recruited in the same city. To be included, they were not allowed to have any cognitive complaints or any significant neurological or psychiatric illness and they needed to have a well-preserved general cognitive functioning. All controls were assessed with either magnetic resonance imaging (MRI) or computed tomography (CT) of the brain. A careful clinical interview, together with an assessment of global function (Mini-Mental State Examination, MMSE), delayed recall (Alzheimer’s Disease Assessment Scale Cognitive Subscale, ADAS Cog, item 3), attention (a quick test of cognitive speed, AQT) and visuospatial and executive function (cube-drawing test and clock test), was done to rule out mild cognitive impairment. Patients diagnosed with AD met the DSM-IIIR criteria for dementia [29] and the criteria for probable AD, as defined by NINCDS-ADRDA [30]. All subjects were assessed by medical doctors with extensive experience in cognitive disorders. For all patients and controls, blood plasma and CSF samples were obtained at some point between 8 a.m. and 12 a.m. White matter lesions (WMLs) were quantified in individuals who had undergone CT scans of their brains, using the ARMMC (Age-related White Matter lesions) scale [31]. All controls provided written informed consent to participate in this study. Due to the retrospective study design, written consent was not possible from the AD patients. Data and CSF from those patients were collected as part of a clinical routine investigation and in conjunction with this they gave oral informed consent for future use of their banked CSF samples for research. This fact was documented in the patients’ medical records. All patients were later on instructed to withdraw their permission, had they changed their minds, as instructed in local press advertisements. The design of this study has been approved by the Local Ethics Committee of Lund University, Sweden and the study procedure was conducted in accordance with the Helsinki Declaration.

### CSF samples and IGF measurements

CSF was collected in polypropylene tubes and mixed gently to avoid gradient effects. All samples were centrifuged within 30 min at +4°C at 2000 *g* for ten minutes to remove cells and debris. Samples were stored in aliquots at -80°C pending biochemical analysis. The procedure followed The Alzheimer’s Association Flow Chart for LP and CSF sample processing [32]. Commercial kits from Mediagnost GmbH (Reutlingen, Germany), were used for all analyses of the IGF system. Levels of IGF-II and IGFBP-2 were analyzed using a sandwich enzyme-linked immunosorbent assay (ELISA), while radioimmunoassays (RIAs) were used for IGF-I and IGFBP-3. Spike analyses were performed and test subject samples were diluted in accordance with the kits’ manuals. The mean correctional values (CVs) for IGF-I, IGF-II, IGFBP-2 and IGFBP-3 in blood plasma was 8.6% (standard deviation, SD = 6.4%), 3.1% (SD = 3.2%), 2.4% (SD = 2.3%) and 4.1% (SD = 3.2%) respectively. The CV in CSF for IGF-I. IGF-II, IGFBP-2 and IGFBP-3 was 10.7% (SD = 9.8%), 1.7% (SD = 1.7%), 2.5% (SD = 2.7%) and 3.7% (SD = 3.5%) respectively.

The levels of β-amyloid_1–42_ (Aβ42), tau and tau phosphorylated at Thr181 (P-tau) were determined using xMAP technology as described [33].

### Statistical analysis

The statistical analyses were made with IBM SPSS for Macintosh, version 19.0.0 (IBM Corp., Armonk, NY, USA). Mann–Whitney nonparametric *U* tests were used for comparing age and MMSE scores between the two groups, while a Pearson’s **χ**^2^ test was used for comparing gender distribution and vascular risk factors. To adjust for the potentially confounding effects of age, continuous variables were log-transformed to obtain a normal distribution, before a general linear model analysis of covariance (ANCOVA) was performed for each biomarker, with age included as a co-variate in the analyses. We then performed ANCOVA analyses for each biomarker, with both age and body mass index (BMI) included as co-variates. Even though the gender distribution did not differ in a statistically significant way between the two groups, we also performed an ANCOVA analyses for each biomarker, with age, BMI and gender included as co-variates. Age, gender, and IGF and IGFBP levels were available in all cases, but BMI was only available in 47 controls and 88 patients with AD. Because of the high CVs for the analyses of IGF-I, we also excluded cases with a CV >20% in a separate analysis. Spearman’s correlation coefficient r_s_ was determined for bivariate correlation analyses.

## Results

The demographic data and measurements of IGF-I, IGF-II, IGFBP-2 and IGFBP-3 levels in blood plasma and CSF are shown in Table 1. Whereas no statistically significant difference was found in gender distribution, the patients with AD were slightly older (p < 0.05) than the controls (Table 1).

**Table 1:** Demographics, vascular risk factors and IGF levels in plasma and CSF

	**Controls (n = 72)**	**AD (n = 92)**
Gender (male/female)	16/56	32/60
Age (years)	75 ± 7	76 ± 7^a^
MMSE at baseline	29 ± 2	19 ± 4^b^
Body Mass Index (BMI)	27 ± 5	24 ± 4^b^
Arterial hypertension (yes/no)	19/53	27/65
Diabetes mellitus (yes/no)	6/66	9/83
Hyperlipidemia (yes/no)	18/54	14/77
Atherosclerotic disease (yes/no)	9/61	13/78
Previous stroke (yes/no)	0/72	0/92
CSF tau	95 ± 50	173 ± 97^b^
CSF P-tau	31 ± 18	130 ± 74^b^
CSF Aβ42	260 ± 77	157 ± 41^b^
CSF IGF-I	0.60 ± 0.20	0.62 ± 0.25
CSF IGF-II	41 ± 7	45 ± 8^a^
CSF IGFBP-2	122 ± 23	136 ± 27^a^
CSF IGFBP-3	18 ± 6	21 ± 8
CSF IGF-I/IGFBP-3	0.035 ± 0.009	0.032 ± 0.009
Plasma IGF-I	99 ± 38	92 ± 39
Plasma IGF-II	680 ± 105	619 ± 114^b^
Plasma IGFBP-2	483 ± 234	632 ± 354^b^
Plasma IGFBP-3	2.3 ± 0.65	2.0 ± 0.57^a^
Plasma IGF-I/IGFBP-3	44 ± 19	46 ± 20

Data are presented as means ± standard deviations, or counts. CSF and blood plasma biomarker levels are given in ng/ml, except for plasma IGFBP-3, which is given in μg/ml. CSF tau, CSF p-Tau and CSF Aβ42 are given in ng/L. The BMI, given in kg/m^2^, was available for 88 patients with AD and 47 controls. ^a^p < 0.05 vs. controls; ^b^p < 0.005 vs. controls. Data were not available for all individuals regarding hyperlipidemia and atherosclerotic disease. In this study, previous myocardial infarction, angina pectoris or stenosis of carotid arteries were used as markers for atherosclerotic disease. For the purpose of this table, an ANCOVA analysis adjusted for age was used for all biomarkers in blood plasma and CSF.

**Figure 1:**
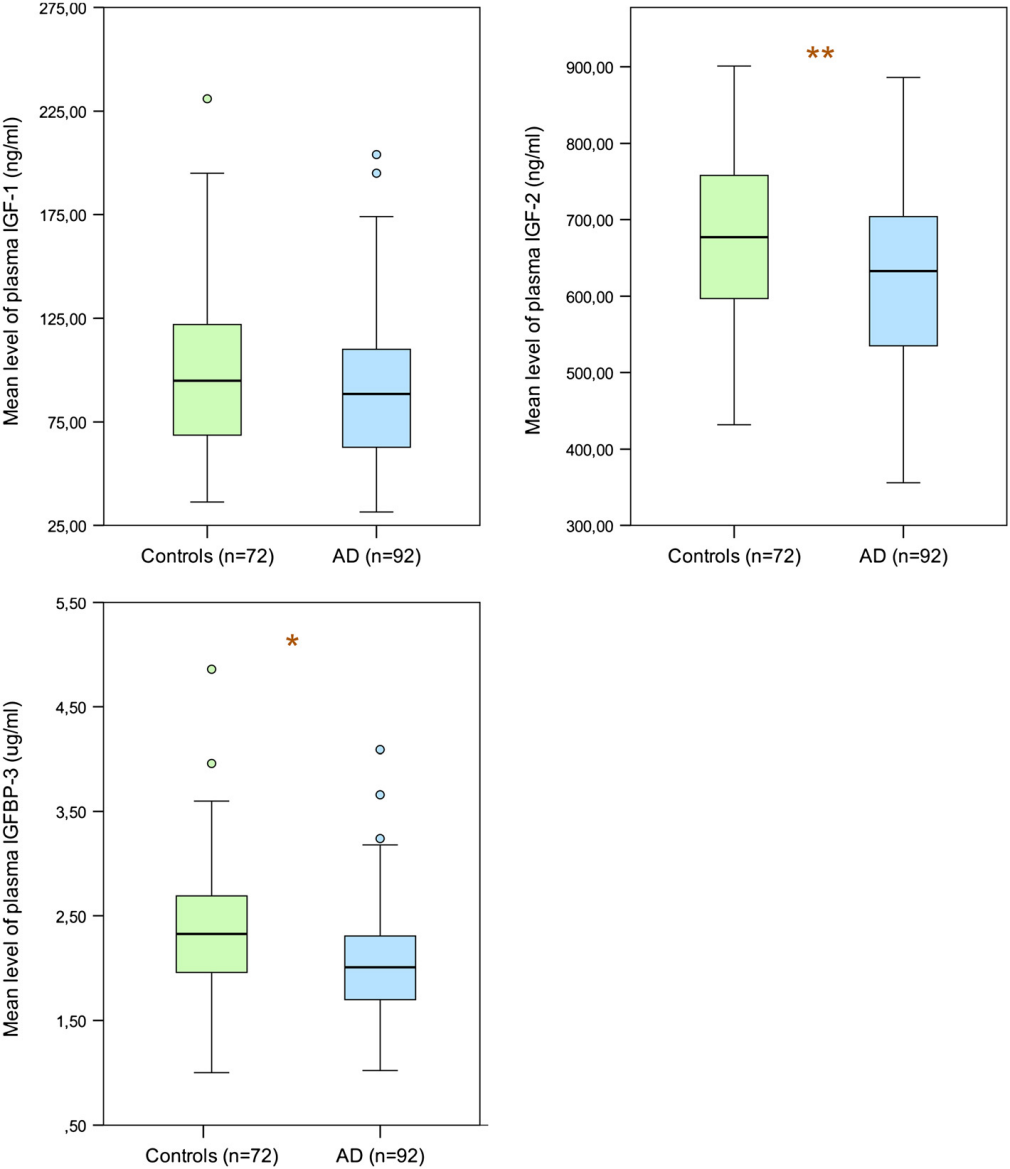
**Concentrations of IGF-I, IGF-II and IGFBP-3 (the most abundant carrier in the bloodstream) in plasma.** The lower, upper and middle lines correspond to the 25^th^ percentile, 75^th^ percentile and median, respectively. The whiskers at the top and bottom extend between the 95^th^ and the 5^th^ percentiles. The circles represent outliers. *p < 0.05 and **p < 0.005.

**Figure 2:**
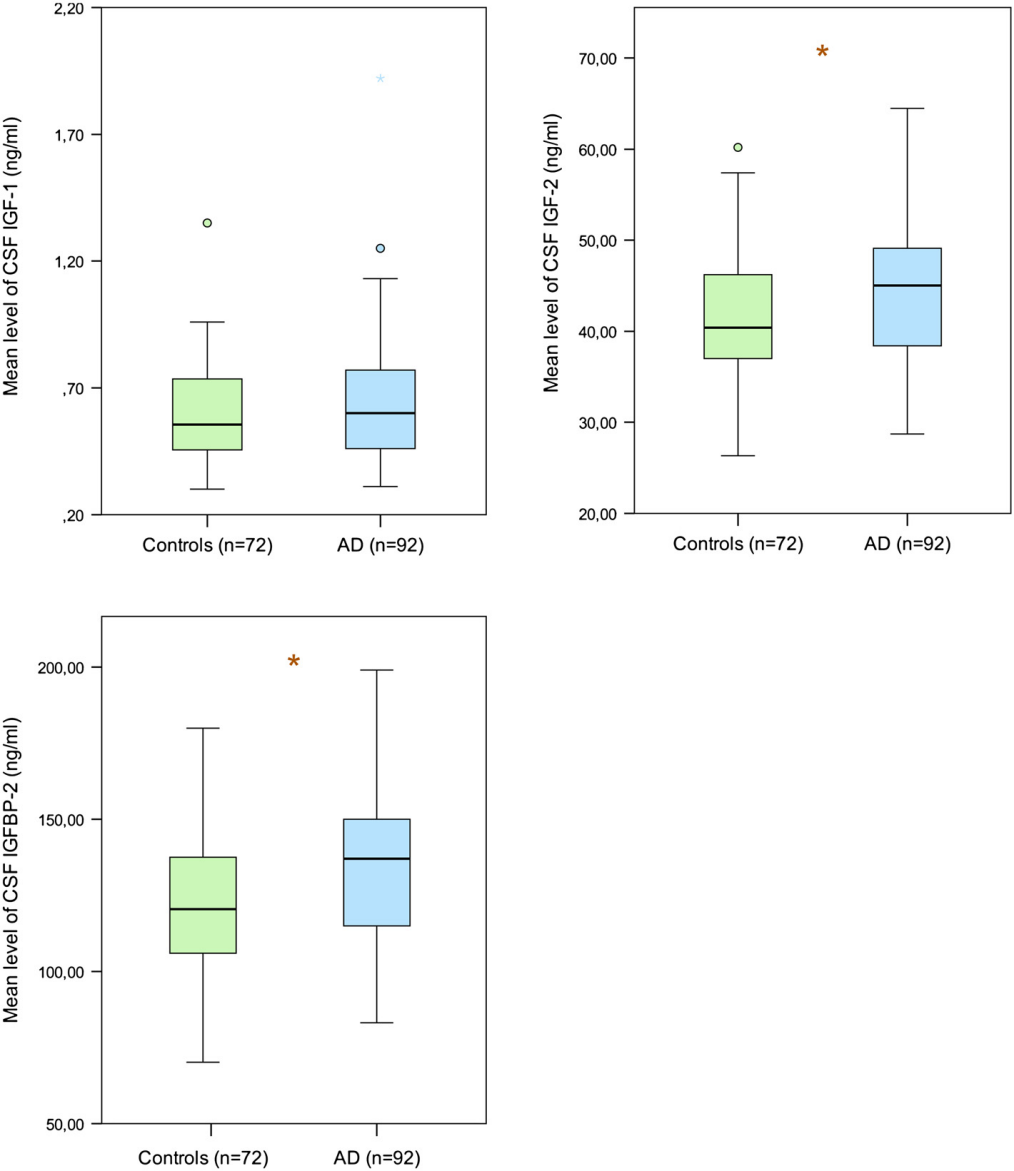
**Concentrations of IGF-I, IGF-II and IGFBP-2 (the most abundant carrier in the intrathecal space) in CSF.** The lower, upper and middle lines correspond to the 25^th^ percentile, 75^th^ percentile and median, respectively. The whiskers at the top and bottom extend between the 95^th^ and the 5^th^ percentiles. The circles represent outliers. *p < 0.05.

In blood plasma, levels of IGF-II were significantly decreased in patients with AD (p < 0.005), even after adjusting for age (Table 1; Figure 1). The level of the main IGF-binding protein in plasma (i.e., IGFBP-3) was also reduced (p < 0.05), but the levels of IGFBP-2 were significantly higher (p < 0.005). In CSF, levels of IGF-II and the main IGF-binding protein in CSF (i.e. IGFBP-2) were significantly higher in patients with AD, even after adjusting for age in statistical analyses (p < 0.05) (Table 1; Figure 2).

The results presented above remained fairly unchanged when adjusting for both age and BMI. In CSF, the difference in levels of IGF-II, IGFBP-2 and IGFBP-3 reached statistical significance (p < 0.05). However, the level of IGF-I in CSF still did not differ between the two groups. In blood plasma, differences in levels of IGF-II and IGFBP-3 reached statistical significance (p < 0.005). Levels of IGF-I and IGFBP-2 in blood plasma did not differ between the two groups.

When adjusting for age, BMI and gender (even though gender did not differ between patients and controls in a significant way), only IGF-II and IGFBP-3 in blood plasma reached statistical significance (p < 0.05). In CSF samples, IGF-II and IGFBP-2 very nearly reached a significance level of p = 0.05 (data not shown). It is possible that this arose from a loss of statistical power due to small sample sizes. Nevertheless, we also analyzed differences between patients with AD and controls in men and women separately, still adjusting for age and BMI. While the sample sizes were then even smaller, it is interesting to note that while we found no differences among the women, men had significantly reduced levels of IGF-II and IGFBP-3 in blood plasma (p < 0.05 and p < 0.001, respectively).

In light of evidence linking the drug galantamine to an increase in hippocampal IGF-II mRNA levels in a mouse model [22], we also excluded patients with AD who had already started treatment with any type of acetylcholinesterase inhibitor at the time of collection of CSF (10 individuals). This did not change our findings in any significant way (data not shown).

Levels of IGF-I in blood plasma or CSF did not differ significantly between the patients with AD and controls. Because of the high CV values for IGF-I, we also excluded cases with CV values above 20% for IGF-I in blood plasma or CSF, but differences between the two groups still did not reach a statistical significance (data not shown).

### Correlations

The correlations between biomarkers in CSF and MMSE in all study participants are shown in Table 2.

**Table 2:** Correlations between biomarkers in CSF and MMSE in patients with AD and controls combined

	**CSF Aβ42**	**CSF Tau**	**CSF P-Tau**	**MMSE**
CSF IGF-I	0.253^a^	0.191	0.116	-0.005
CSF IGF-II	0.058	0.161	0.155	-0.178^a^
CSF IGFBP-2	0.034	0.463^b^	0.374^b^	-0.250^b^
CSF IGFBP-3	0.060	0.308^b^	0.266^a^	-0.143
MMSE	0.374^b^	-0.442^b^	-0.567^b^	1.000

Numbers are Spearman correlation coefficients (r_s_). ^a^p < 0.05; ^b^p < 0.005. Abbreviations: *CSF* cerebrospinal fluid, *IGF* insulin growth factor, *IGFBP* insulin growth factor binding protein, *MMSE* Mini-Mental State Examination.

In patients with AD, the CSF levels of tau and P-tau correlated positively with CSF levels of IGFBP-2 (p < 0.05, r_s_ = 0.380 and 0.398, respectively). Levels of P-tau also correlated positively with the CSF levels of IGF-I (p < 0.05, r_s_ = 0.253) and CSF levels of IGFBP-3 (p < 0.005, r_s_ = 0.322) (Table 3). In healthy controls there was a positive correlation between tau and all analyzed IGF components in CSF (Table 4).

**Table 3:** Correlations between biomarkers in CSF and MMSE in patients with AD

	**CSF Aβ42**	**CSF Tau**	**CSF P-Tau**	**MMSE**
CSF IGF-I	0.220	0.146	0.253^a^	0.096
CSF IGF-II	0.155	0.050	0.183	0.034
CSF IGFBP-2	0.177	0.380^b^	0.398^b^	-0.075
CSF IGFBP-3	0.294	0.212	0.322^a^	0.070
MMSE	-0.079	-0.208	-0.100	1.000

Numbers are Spearman correlation coefficients (r_s_). ^a^p < 0.05; ^b^p < 0.005. Abbreviations: *CSF* cerebrospinal fluid, *IGF* insulin growth factor, *IGFBP* insulin growth factor binding protein, *MMSE* Mini-Mental State Examination.

**Table 4:** Correlations between biomarkers in CSF and MMSE in healthy controls

	**CSF Aβ42**	**CSF Tau**	**CSF P-Tau**	**MMSE**
CSF IGF-I	0.195	0.523^a^	0.199	-0.092
CSF IGF-II	-0.113	0.414^a^	0.155	-0.111
CSF IGFBP-2	-0.014	0.618^b^	0.247	-0.12
CSF IGFBP-3	-0.030	0.547^a^	0.194	-0.080
MMSE	-0.085	0.164	-0.095	1.000

Numbers are Spearman correlation coefficients (r_s_). ^a^p < 0.05; ^b^p < 0.005. Abbreviations: *CSF* cerebrospinal fluid, *IGF* insulin growth factor, *IGFBP* insulin growth factor binding protein *MMSE* Mini-Mental State Examination.

We did not find any significant correlations between CSF and blood plasma levels in any of the four IGF-system components analyzed (data not shown. Further, we found no correlations between MMSE scores and CSF or blood plasma levels of any of the four IGF-system components in the AD group (Table 3).

When comparing IGFs with WMLs there were no significant correlations between either IGF-I or IGF-II and WMLs (data not shown).

## Discussion

Here we found significantly lower blood plasma levels of IGF-II and IGFBP-3 (the main IGF-binding protein in plasma) in patients with AD. In the CSF of these patients, we also found higher levels of both IGF-II and IGFBP-2 (the main IGF-binding protein in CSF). When adjusting for age, gender and BMI, only levels of IGF-II and IGFBP-3 in blood plasma differed between the two groups, possibly because of significantly lower levels of these two proteins in men with AD. Furthermore, there were significant correlations between components of the IGF-system and tau in healthy controls and between components of the IGF system and P-tau in patients with AD.

There were several limitations of this study. The apolipoprotein E ϵ4 allele is a known risk factor for AD, but was not analyzed for the controls in this material. Nutritional status is known to affect the IGF system [34]. Unfortunately, we only obtained BMI data on a subset of patients with AD and controls. Also, pre-albumin and albumin, as markers of nutritional status were not analyzed.

We mainly focused on investigating changes in IGF-II levels in blood plasma and CSF in patients with AD. To the best of our knowledge, only one previous study has explored IGF-II levels in the CSF of such patients. In an analysis of 10 patients and 10 controls, Tham et al. [35] found no difference in CSF levels of IGF-I, while there was a significant elevation of IGF-II in CSF, which is in accordance with our present findings. They also found significantly elevated CSF levels of two binding proteins, which they believed to be IGFBP-2 and IGFBP-6, in part matching our result.

The existing literature on changes in CSF or plasma levels of IGF-I in patients with AD is divergent and often contradictory [17,36,37]. In a large multicentre study, Duron et al. found significantly lower levels of IGF-I and IGFBP-3 in the blood plasma of male patients with AD [38]. We found similar results for the IGFBP-3 level in blood plasma. On the other hand, whereas Johansson et al. found elevated levels of IGF-I in serum, they also found significantly higher serum levels of IGFBP-3 [12].

Much effort has been invested in developing treatment strategies targeting the hypothetically skewed nature of amyloid homeostasis—most notably immunomodulatory therapies aimed at increasing the clearance of Aβ42. To date, this approach has not been able to limit the progress of the disease, even though the Aβ42 burden of the brain has been shown to diminish [39]. Perhaps these treatment studies have been performed with patients too far along in the course of the disease, which might explain the discouraging results. However, another possibility is that other pathological mechanisms, in addition to the accumulation of β-amyloid, are important for individuals affected by sporadic AD. For example, an ineffective IGF system that fails to uphold the neuroregenerative and neuroprotective mechanisms necessary for a healthy brain, might contribute to the pathological changes seen in patients with AD. This could be caused by decreased levels of active IGFs [19], or by errors elsewhere in the signaling pathway from the cell receptor to the cell nucleus [40]. The latter would be equivalent to a decreased sensitivity of the brain to IGFs, analogous to an increased insulin resistance. Indeed, insulin and IGF share a high degree of structural and functional homology and each of them bind to–and activate–the receptor of the other molecule [41,42]. An increased insulin resistance of the brain has been implied to play a role in AD [5,21]. Thus, Talbot et al. demonstrated elegantly that patients with AD show a markedly reduced response to insulin in the hippocampus and the cerebellar cortex, accompanied by a greatly reduced response to IGF-I as well. The magnitude of these changes increased progressively, from cognitively normal cases, through patients with mild cognitive impairment, to patients with full-blown AD, regardless of the incidence of diabetes [43]. We found the levels of IGF-II in CSF to be significantly higher in patients with AD, as would be expected if there indeed were an increased resistance to IGFs, thus supporting this hypothesis.

Faulty IGF-binding proteins could also play a role in the development of AD. An over-expression of IGFBP-2 has been shown to reduce postnatal growth in mice, most likely by inhibiting IGF [44]. Our data showed an increase of IGFBP-2 in both plasma and CSF, which might diminish the bioactivity of IGF.

A third possibility could be that the changes in the IGF system is not at all a part of the disease process in patients with AD, but rather a part of the body’s defense against brain damage. Both IGF-I and IGF-II seem to protect against intrinsic and extrinsic cell death stimuli [45]. Thus, inducing traumatic brain damage in mice provoked a brief induction of IGF-I expression and its associated signalling components in the acute post-traumatic period [46]. Also, administration of IGF-I to brain-damaged rats seemed to ameliorate neurobehavioural dysfunction [47]. In humans, increasing circulating levels of IGF-I by adminestering GH, seemed to improve disabilities after traumatic brain damage, including improved cognitive functions [48]. In the present study the levels of tau–an established marker for neuronal damage [49]–correlated positively with the levels of IGF-I, IGF-II, IGFBP-2 and IGFBP-3 in healthy controls, suggesting that the levels of these IGF system components may indeed increase with neuronal damage–possibly as a neuroprotective response.

In AD, levels of P-tau are generally increased when compared to healthy controls, as a result of the higher phosphorylated state of tau in the brain, with more NTFs [50]. The NTF burden of the brain has in turn been shown to correlate with the degree of neuronal loss in AD [51]. In the present study, the levels of P-tau in patients with AD were positively correlated with those of IGF-I, IGFBP-2 and IGFBP-3, albeit with correlation coefficients less than 0.4. This suggests an association between levels of IGF system components and P-tau, which supports the theory that the levels of the IGF system components increase as a response to neuronal damage also in AD.

## Conclusions

In conclusion, we found changes in the IGF-related system in patients with AD, including altered levels of IGF-II in CSF and blood plasma. There were also significant correlations between IGF system components and established biomarkers for AD in the CSF. However, further investigations are necessary to unravel the mechanisms behind the altered IGF-II levels in patients with AD, which might provide important clues to the nature of AD, as well as potential new treatment strategies.

## Competing interests

The authors declare that they have no competing interests.

## Authors’ contributions

JH participated in the study design, performed the statistical analyses and drafted the manuscript. OH participated in the study design and the statistical analyses and critically revised the manuscript. Both KN and LM made major contributions to the acquisition of data and have critically revised the manuscript. All authors have given final approval of the version to be published.

## Pre-publication history

The pre-publication history for this paper can be accessed here:

http://www.biomedcentral.com/1471-2377/14/64/prepub
